# Future Perspectives in the Diagnosis and Treatment of Sepsis and Septic Shock

**DOI:** 10.3390/medicina58070844

**Published:** 2022-06-24

**Authors:** Irene Karampela, Paraskevi C. Fragkou

**Affiliations:** 1 Second Department of Critical Care, Attikon General University Hospital, Medical School, National and Kapodistrian University of Athens, 12462 Athens, Greece; 2 First Department of Critical Care Medicine and Pulmonary Services, Evangelismos Hospital, Medical School, National and Kapodistrian University of Athens, 10676 Athens, Greece; evita.fragou@gmail.com

Sepsis, defined as life-threatening organ dysfunction caused by a dysregulated host response to infection, represents the primary cause of death due to infection [1]. Unless diagnosed and treated early, sepsis can lead to septic shock, multiple organ failure and death [2]. The global burden of sepsis has substantially increased during the last decades. According to a recent study, the global estimate of sepsis incidence was 49 million cases, while sepsis-related deaths reached 11 million in 2017, accounting for almost 20% of all-cause deaths globally [3]. Moreover, hospital-acquired infections leading to sepsis represent up to one-quarter of all hospital-treated sepsis cases [4]. Based on a recent meta-analysis, the hospital mortality rate for sepsis is estimated to be almost 27%, while for sepsis patients treated in the intensive care unit, the mortality rate is as high as 42% [5].

Prompt diagnosis and appropriate treatment of sepsis are of utmost importance and key to survival [6]. However, routinely used biomarkers, such as C-reactive protein and procalcitonin, have shown moderate diagnostic and prognostic value. Of note, the recent consensus definition for sepsis is based on clinical criteria, implying the paucity of reliable sepsis biomarkers. The new diagnostic criteria also incorporate the use of the SOFA score, a composite prediction tool, which is derived by a combination of clinical signs and biomarkers of organ dysfunction, leaving aside classic inflammatory biomarkers [1]. This further highlights the lack of a gold standard diagnostic test for sepsis. Therefore, the study of novel biomarkers that may facilitate the early diagnosis of sepsis continues to be a challenging research field [7].

Numerous molecules, such as cytokines and chemokines, involved in the inflammatory cascade in sepsis are being studied to identify better-performing biomarkers [7,8]. In this context, certain adipokines (cytokine-like molecules derived from the adipose tissue) have been recently shown to have diagnostic value in sepsis, while their kinetics early in sepsis were found to be independent predictors of sepsis outcome in prospective observational studies [9,10,11,12,13,14]. Moreover, advances in proteomics have supported the investigation of a high number of molecules (the so-called secretome) that are produced and secreted during sepsis [15,16]. Of note, progress in bioinformatics and artificial intelligence has shown promising results in the development of breakthrough diagnostic tools by combining multiple biomarkers identified by experimental and clinical studies [17]. Additionally, a machine learning approach using large data sets may improve the performance of currently used screening models for the early and accurate identification of patients with sepsis [18]. Research in the field of omics technologies and the use of high-dimensional data are expected to pave the ground for unraveling the pathophysiologic mechanisms of sepsis, promoting diagnostics and identifying new therapeutic targets to guide future therapies and precision medicine [17,19].

Although the fundamental principles of the treatment of sepsis and septic shock (initial resuscitation, infection control, and organ support) remain unchanged, there are still several gaps regarding various aspects of clinical management. As highlighted in the recent Surviving Sepsis Campaign international guidelines, the evidence designating the type, dose and timing of resuscitation fluids is still of low quality, being mainly observational [6]. Additionally, reliable biomarkers for monitoring the response to treatment, as well as clear therapeutic goals, are controversial due to the paucity of high-quality evidence such as large randomized controlled trials.

Regarding the hemodynamic management of septic shock, there is a great interest in the type and timing of various inotropes and vasopressors besides norepinephrine. The role of vasopressin and its analogues, such as selepressin, as well as angiotensin II, is yet to be determined [20]. Moreover, the use of short-acting β-blockers, such as esmolol and landiolol, to control ventricular rate in septic shock has shown a beneficial effect on outcome [21]. 

Regarding corticosteroids, there is not yet adequate evidence to support their routine use in the treatment of sepsis and septic shock [22]. However, their beneficial use in the treatment of severe acute respiratory syndrome coronavirus 2 (SARS-CoV-2) infection brought them to the front, renewing research interest in their anti-inflammatory properties. Moreover, the coronavirus disease 2019 (COVID-19) pandemic urged researchers to explore the therapeutic value of immune-based treatment options to block the cytokine storm in SARS-CoV-2-related sepsis, with promising results [23,24]. Immunotherapeutic agents targeting immune mediators of host defense such as corticosteroids, anti-cytokine agents and kinase inhibitors may prove useful in blocking hyperinflammation in sepsis [24]. However, as the host response to infection can vary substantially between patients, these treatments warrant investigation using an individualized approach based on the immunological profile of patients [25,26].

Blood purification techniques (hemoperfusion or hemadsorption), aiming at the extracorporeal cytokine removal and reducing endotoxin activity, have shown potential as adjunctive treatments for sepsis. Recent advances in the development of new absorptive materials and hemoperfusion devices have motivated clinical studies in patients with sepsis, showing a significant decrease in circulating cytokine and endotoxin concentrations with favorable hemodynamic effects [27]. However, evidence from the clinical application of hemoperfusion is limited and controversial [28]. Large randomized controlled trials are needed to investigate the therapeutic role of blood purification modalities in sepsis.

Basic research has made substantial progress during the last decades contributing to our better understanding of the pathophysiologic mechanisms of sepsis, and clinical studies have led to important therapeutic advances and outcome improvements. However, as stated above, there are still many gaps to be filled and many quandaries regarding diagnostics and therapeutics of sepsis and septic shock. New strategies are urgently needed to combat the heavy burden of sepsis.
